# The impact of physical activity levels and cardiorespiratory fitness on heart rate variability in overweight and obese college students: a cross-sectional study

**DOI:** 10.7717/peerj.20612

**Published:** 2026-01-19

**Authors:** Meihua Su, Jiajing Wang, Fengxun Lin, Zange Lin, Jianming Chen

**Affiliations:** 1 Physical Education Institute, Jimei University, Xiamen, China; 2 Psychological Counseling Center, Jimei University, Xiamen, China

**Keywords:** Obesity, College students, Physical activity, Heart rate variability, Cardiorespiratory fitness

## Abstract

**Objective:**

This study aimed to examine the impact of varying physical activity (PA) intensities on heart rate variability (HRV), to explore the relationships among cardiorespiratory fitness (CRF), body composition, and HRV, and to further characterize sex-specific HRV patterns in overweight and obese college students.

**Methods:**

A total of 184 overweight or obese college students were enrolled in this cross-sectional study. Participants were categorized into low, medium, or high intensity PA groups using the International Physical Activity Questionnaire–Short Form (IPAQ-SF). Body fat percentage and muscle mass were assessed using bioelectrical impedance analysis, while CRF was estimated from maximal oxygen uptake (VO_2_max) derived from a cycle ergometer test. Resting HRV indices were obtained under standardized conditions. Differences in body composition, CRF, and HRV were analyzed across groups. Multiple linear regression models were further employed to identify independent predictors of HRV.

**Results:**

High-intensity PA independently predicted log(standard deviation of normal RR intervals (SDNN)) (b = 0.40, 95% CI [0.12–0.67], *P* = 0.005) and log(root mean square of successive differences (RMSSD)) (b = 0.48, 95% confidence interval (CI) [0.13–0.83], *P* = 0.007), while medium-intensity PA also showed a significant effect on log(SDNN) (b = 0.22, 95% CI [0.04–0.39], *P* = 0.014). Female participants exhibited higher log(high-frequency component (HFn)) (b = 0.25, 95% CI [0.11–0.39], *P* < 0.001) and lower log(low frequency/high frequency (LF/HF)) (b = −0.39, 95% CI [−0.62 to −0.17], *P* < 0.001) compared with males. VO_2_max was not significantly associated with most HRV indices, remaining significant only for log(SDNN) (b = 0.46, 95% CI [0.02–0.90], *P* = 0.042).

**Conclusions:**

Medium-to-high intensity PA independently predicted favorable HRV indices. Female students demonstrated higher HRV levels than males, suggesting sex specific. The association between PA and autonomic regulation appeared stronger than that between VO_2_max and HRV.

## Introduction

Overweight (OW) and obesity (OB) have become major global public health concerns, with their prevalence steadily increasing among young adults, including college students (Powell-Wiley et al., 2021; Shafiee et al., 2024). These conditions not only contribute to metabolic disturbances but also markedly elevate the risk of cardiovascular diseases (CVD) (Csige et al., 2018; Powell-Wiley et al., 2021). Emerging evidence indicates that autonomic nervous system (ANS) dysfunction is a key early marker of obesity-related cardiovascular risk. Heart rate variability (HRV), a non-invasive indicator reflecting autonomic activity and regulatory capacity, has been widely employed to assess cardiovascular health (Gitler et al., 2025; Strüven et al., 2021; Williams et al., 2019). Lower HRV typically indicates impaired parasympathetic activity and/or heightened sympathetic dominance, thereby increasing the likelihood of adverse cardiovascular events (Khan, Lip & Shantsila, 2019; Pagkalos et al., 2008).

Physical activity (PA) represents a cornerstone lifestyle intervention for improving health outcomes in individuals with OW or OB. Prior studies consistently demonstrate that regular PA enhances autonomic function, as evidenced by improvements in HRV (Kang, Ko & Baek, 2016; May et al., 2017; Pope et al., 2020; Tornberg et al., 2019). Both aerobic and resistance exercise modalities have been reported to positively influence HRV parameters, although the effects vary according to exercise type, intensity, and duration (Zhang, Bi & Luo, 2025). A 2023 systematic review including 14 studies of overweight or obese adults reported medium associations between habitual PA and HRV, particularly for time- and frequency-domain indices, highlighting the role of PA in mitigating autonomic dysfunction (Kumar et al., 2023).

Among children and adolescents with obesity, exercise interventions enhanced HRV, particularly in time-domain measures of parasympathetic activity. However, evidence specifically addressing college-aged populations remains limited (Bitencourt et al., 2024). A 2022 systematic review focusing on overweight and obese college students reported that PA-based interventions improved general health outcomes, yet only a subset of studies assessed autonomic outcomes such as HRV, and the effects were inconsistent across different exercise intensities (Pfisterer et al., 2022).

Sedentary behavior is increasingly prevalent among college students with OW or OB, further exacerbating obesity-related cardiometabolic risks. While numerous studies in the general population indicate beneficial effects of PA on HRV, the specific impact of different PA intensities and potential dose–response relationships within overweight or obese young adults remains underexplored. For instance, a 2024 study in young adults demonstrated a dose-dependent relationship between exercise intensity and HRV, but highlighted the paucity of data specifically in obese subgroups, underscoring the need for more targeted research (Su et al., 2024).

Cardiorespiratory fitness (CRF), typically expressed as maximal oxygen uptake (VO_2_max), has been proposed as a mediator of the PA and HRV relationship. Higher CRF is generally associated with greater HRV. However, the independent association between CRF and HRV after adjusting for confounding factors such as PA level, body composition, age, and sex has not been fully elucidated among overweight or obese young adults (Mabe-Castro et al., 2024). Furthermore, HRV exhibits notable sex differences, with females generally displaying higher parasympathetic activity and lower sympathovagal balance ratios compared with males (Carnevali et al., 2023; Hill et al., 2021; Zhu et al., 2025). Whether these sex-related differences persist independently in overweight or obese college students, or are mediumd by obesity-related factors, remains unclear.

Body composition also plays a critical role in autonomic regulation. Increased visceral adiposity, higher waist-to-hip ratio, and overall body fat percentage are often associated with reduced HRV and heightened sympathetic dominance, which may elevate cardiovascular risk (Cvijetic et al., 2023; Grassi et al., 2007; Triggiani et al., 2019). Despite these associations, few studies have simultaneously accounted for PA, CRF, sex, and body composition when examining HRV in college-aged individuals with OW or OB.

Given the interplay between PA, CRF, sex, and body composition, clarifying the independent contributions of each factor to HRV is critical for optimizing cardiovascular risk assessment and designing effective interventions. Understanding these relationships may inform tailored PA programs that maximize autonomic benefits, particularly in populations at elevated cardiometabolic risk.

## Materials and Methods

### Sample size calculation

This study aimed to investigate the relationships among physical activity, cardiorespiratory fitness, and heart rate variability in overweight and obese college students in Xiamen City. Based on previous research (Lavie et al., 2019), a two-sided test was applied with an alpha (α) level of 0.05, an estimated standard deviation of 30, and a permissible error of 5. Using the relevant formula, the calculated sample size was *N* = 157 participants. Considering a questionnaire response rate of 90%, at least 175 participants were required. Ultimately, a total of 184 valid samples were included.

### Participants

Participants were classified as overweight or obese according to their body mass index (BMI). The Chinese national criteria were applied: overweight (24.0 kg/m^2^ ≤ BMI ≤ 27.9 kg/m^2^) and obesity (BMI ≥ 28.0 kg/m^2^) (Zhou & the Cooperative Meta-analysis Group of Working Group on Obesity in China, 2002). These criteria differ from the World Health Organization (WHO) definitions (overweight: BMI ≥ 25 kg/m^2^; obesity: BMI ≥ 30 kg/m^2^). All participants provided written informed consent voluntarily. Exclusion criteria included: presence of cardiovascular, respiratory, metabolic (*e.g*., diabetes), or endocrine disorders (*e.g*., thyroid dysfunction) affecting HRV; use of medications influencing the autonomic nervous system or HRV; smoking or alcohol consumption; weight fluctuations >5 kg within the past 6 months. This study has been approved by the Ethics Committee of Jimei University, with the approval number JMU202407065.

### Physical activity level

PA was evaluated using the Chinese short version of the International Physical Activity Questionnaire (IPAQ-SF), which has demonstrated good validity and reliability (Ács et al., 2021; Lee et al., 2011). To accurately capture habitual activity patterns, participants first completed a 2-week open activity log, followed by the formal IPAQ-SF assessment.

Participants were categorized into three groups based on their physical activity levels quantified by metabolic equivalents (METs): low (<3 METs), medium (3.1–6 METs), and high (≥6 METs) (Deng et al., 2008). The total PA volume was calculated according to the IPAQ manual: PA volume (MET-min/week) = MET value × duration (min/day) × frequency (days/week) (Oliveira et al., 2022).

### Body composition

Body composition was measured using a bioelectrical impedance analyzer (TANITA MC-180, Tokyo, Japan). All measurements were performed after device calibration. Parameters included height, body weight, body fat percentage (BFP%), skeletal muscle mass (SMM), visceral fat content (VFC), and waist-to-hip ratio (WHR).

### Cardiorespiratory fitness

CRF was assessed using the Astrand–Rhyming submaximal test, estimating maximal oxygen uptake (VO_2_max) based on heart rate responses to submaximal workloads. Short-term submaximal exercise testing provides valuable, non-invasive screening for cardiovascular risk in obese individuals (Ferrari et al., 2025). Participants wore a Polar H10 heart rate sensor and performed an incremental cycling test on an ergometer (ERGOSANA XC-1000, Bitz, Germany). The protocol included a 3-min warm-up at 50 W, followed by 25 W increments every 2 min at 60 revolutions per minute until voluntary exhaustion (≥85% of age-predicted HRmax or RPE ≥ 17). After the test, cardiac functional capacity, absolute VO_2_max, and relative VO_2_max were estimated using the ERGOSANA XC-1000 cycle ergometer’s built-in algorithm, which is proprietary and calculates values based on heart rate, power output, and exercise duration. Forced vital capacity (FVC) was measured using a spirometer (HHTC200-FH, Huaxia Huihai, Guangzhou, China). Participants performed three maximal inspiratory–expiratory maneuvers; the highest value was used for analysis.

### Heart rate variability assessment

HRV measurements were conducted in a quiet, temperature-controlled, well-lit room. Participants were positioned supine and fitted with a Polar H10 heart rate strap connected to Kubios HRV software. R–R intervals were recorded continuously for 5 min under resting conditions (Munoz et al., 2015).

In overweight or obese populations, 5-min HRV recordings may not fully capture autonomic complexity due to frequent autonomic dysfunction, potentially reducing signal sensitivity. To minimize this limitation, a standardized pre-test protocol was applied, including a 15-min quiet rest and detailed health screening (*e.g*., exclusion of cardiovascular disease, medication use, or sleep disorders).

### Statistical analysis

All data underwent statistical analysis using SPSS 26.0.Shapiro–Wilk tests assessed data normality. HRV and CRF data were skewed; therefore, both HRV and maximal oxygen uptake (VO_2_max) were log-transformed in the regression models. After transformation, all HRV indices except MeanRR remained non-normally distributed. Accordingly, robust linear regression (RLM) models were employed for all HRV parameters except MeanRR, while conventional linear regression was used for MeanRR.

Normally distributed variables were expressed as mean ± standard deviation (SD), non-normally distributed variables as median (M) and interquartile range (Q_1_, Q_3_), and categorical variables as frequencies and percentages. Differences among PA groups were analyzed using Kruskal–Wallis H tests, followed by Dunn’s *post hoc* test with Holm adjustment. Spearman’s rank correlation was used to evaluate bivariate relationships.

Stepwise multiple linear regression models identified predictors of HRV, including PA, age, body fat percentage (BFP%), VO_2_max_rel, and sex. Sex × PA interaction terms were included to examine moderation effects. Variable selection and multicollinearity assessment were performed prior to multiple regression analysis. Multicollinearity among predictors was evaluated using the variance inflation factor (VIF), and variables with VIF > 5—including BMI, visceral fat, waist-to-hip ratio, absolute VO_2_max, cardiac functional capacity, and vital capacity—were excluded from the model. All remaining predictors had VIF < 5, indicating no significant multicollinearity. Model selection was guided by bidirectional stepwise regression based on the Akaike Information Criterion (AIC), which iteratively adds or removes predictors to identify the model that optimally balances goodness-of-fit and model complexity. Statistical significance was set at two-tailed *P* < 0.05.

## Result

### Baseline characteristics of participants

This study recruited 184 overweight or obese college students (mean age: 21 years; mean height: 170.35 cm). Participants were categorized into an overweight group (*n* = 96) and an obese group (*n* = 88) based on BMI. Table 1 presents their demographic characteristics and PA levels.

**Table 1:** Demographic characteristics of participants.

Variables	Total (*n* = 184)	BMI grouping		Statistic	Effect size	*P*
		Overweight (*n* = 96)	Obesity (*n* = 88)			
Age, M (Q_1_, Q_3_)	21.00 (20.0, 22.0)	21.00 (20.0, 22.0)	20.00 (20.0, 22.0)	*Z* = −2.88	*r* = −0.21	0.004
Height, M (Q_1_, Q_3_)	170.35 (161.9, 177.0)	166.20 (160.0, 175.3)	174.00 (168.0, 179.2)	*Z* = −3.89	*r* = 0.29	<0.001
Weight, M (Q_1_, Q_3_)	81.90 (71.9, 92.1)	72.45 (68.2, 80.9)	92.15 (83.7, 102.2)	*Z* = −9.15	*r* = 0.68	<0.001
Gender, *n* (%)				*χ*² = 21.46	*V* = 34	<0.001
Male	110 (59.8)	42 (43.8)	68 (77.3)			
Female	74 (40.2)	54 (56.3)	20 (22.7)			
PA, *n* (%)				*χ*² = 2.29	*V* = 0.11	0.318
Low	87 (47.3)	49 (51.1)	38 (43.2)			
Medium	76 (41.3)	39 (40.6)	37 (42.0)			
High	21 (11.4)	8 (8.3)	13 (14.8)			

**Note:**

Data are presented as median (Q_1_, Q_3_) for continuous variables and *n* (%) for categorical variables. *Z* and *P* values are based on Mann-Whitney *U* tests comparing overweight *vs*. obesity groups (*N* = total sample size, 184). Effect size *r* = *Z*/ $\sqrt N. \chi^{\it2}$ tests were used for gender and physical activity (PA); effect size: Cramer’s *V*.

Significant differences in age, height, and weight were observed between the groups. The obese group was slightly younger than the overweight group but had significantly greater median height and weight. Gender composition also differed markedly: the overweight group comprised more females (56.25%) than males (43.75%), whereas the obese group was predominantly male (77.27%) compared with females (22.73%).

However, the International Physical Activity Questionnaire–Short Form (IPAQ-SF) revealed no statistically significant differences in PA level distribution (low, medium, high intensity) between BMI groups (χ^2^ = 2.29, *P* = 0.318). This indicates a comparable distribution of PA intensity across overweight and obese participants.

### Inter-group comparison of body composition among overweight and obese college students engaging in varying levels of physical activity

No significant differences were observed in body fat percentage (BFP%), skeletal muscle mass (SMM), or waist-to-hip ratio (WHR) among the low-, medium-, and high-PA groups (all *P* = NS).

Body mass index (BMI) differed significantly only between the low- and high-PA groups, with the low-PA group exhibiting a lower BMI (*P* < 0.05). The comparisons between low- and medium-PA groups, as well as between medium- and high-PA groups, were not statistically significant (*P* = NS).

Visceral fat content (VFC) differed significantly between the medium- and high-PA groups (*P* < 0.05), with the medium-PA group showing lower VFC values. No other pairwise comparisons for VFC reached statistical significance.

### Inter-group comparison of cardiopulmonary fitness levels among overweight and obese college students engaging in different physical activities

**Figure 1:**
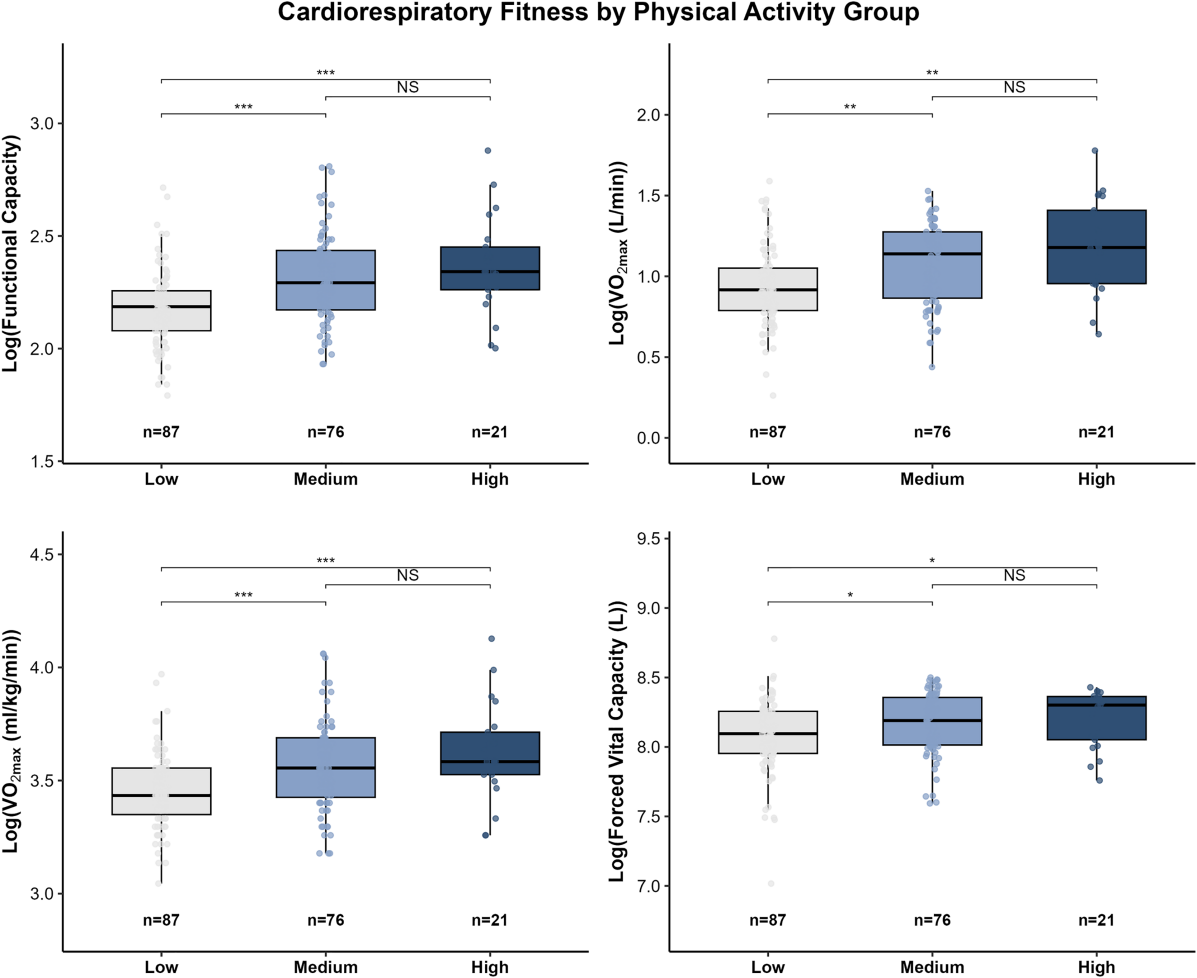
Distribution of cardiorespiratory fitness indicators across physical activity groups. Box plots illustrate the distributions of functional capacity, absolute VO_2_max (L·min^−1^), relative VO_2_max (mL·kg^−1^·min^−1^), and vital capacity (L) among participants with low (*n* = 87), medium (*n* = 76), and high (*n* = 21) physical activity levels. Group differences were assessed using the Kruskal-Wallis test, followed by Dunn’s *post hoc* tests with Holm correction for pairwise comparisons. Outliers are shown as individual points. Significance levels: *P < 0.05; **P < 0.01; ***P < 0.001; NS, not significant (*P* ≥ 0.05).

Between-group differences in cardiorespiratory fitness (CRF) are presented in Fig. 1. Functional capacity differed significantly among PA groups. The low-PA group exhibited lower functional capacity than both the medium-PA (*P* < 0.001) and high-PA groups (*P* < 0.001). No significant difference was observed between the medium- and high-PA groups (*P* = NS).

A similar pattern was observed for maximal oxygen uptake (VO_2_max). Both absolute VO_2_max (L/min) and relative VO₂max (ml/kg/min) were significantly lower in the low-PA group compared with the medium-PA (absolute VO_2_max: *P* < 0.01; relative VO_2_max: *P* < 0.001) and high-PA groups (absolute VO_2_max: *P* < 0.01; relative VO_2_max: *P* < 0.001). Consistent with the functional capacity results, no significant difference in VO_2_max was detected between the medium- and high-PA groups. Similarly, there were between-group differences in vital capacity between the medium-to-high physical activity group and the low physical activity group.

### Heart rate variability differences across physical activity levels in overweight and obese college students

**Figure 2:**
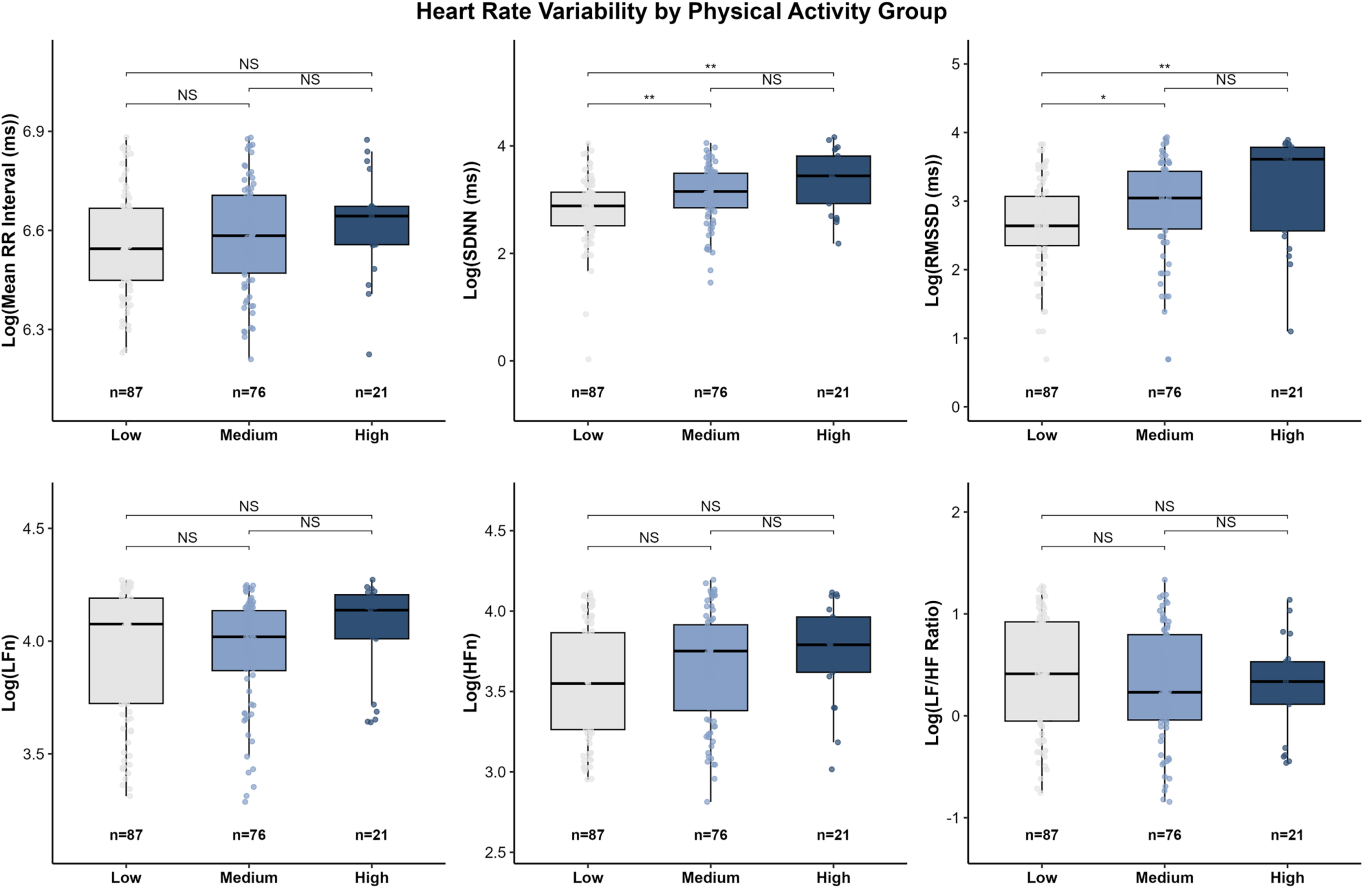
Distribution of log-transformed HRV indices across physical activity groups. Box plots present log-transformed time-domain indices (Mean-RR interval, SDNN, RMSSD) and frequency-domain indices (LFn, HFn, and LF/HF ratio) for participants classified into low (*n* = 87), medium (*n* = 76), and high (*n* = 21) physical activity levels. Overall group differences were analyzed using the Kruskal-Wallis test, followed by Dunn’s *post hoc* tests with Holm correction. Outliers are shown as individual points. Significance levels: *P < 0.05; **P < 0.01; NS = *P* ≥ 0.05.

Between-group differences in heart rate variability (HRV) indices are presented in Fig. 2. Significant differences were observed across PA groups, primarily driven by time-domain measures.

Time-domain measures:

Standard deviation of normal RR intervals (SDNN) differed significantly among PA groups. The low-PA group exhibited the lowest overall autonomic activity, with SDNN values significantly lower than those in the medium-PA (*P* < 0.01) and high-PA groups (*P* < 0.001). No significant difference was found between the medium- and high-PA groups (*P* = NS).

Root mean square of successive differences (RMSSD), an indicator of parasympathetic tone, also showed significant group differences. RMSSD values were lower in the low-PA group compared with the medium-PA (*P* < 0.05) and high-PA groups (*P* < 0.01). Although the high-PA group displayed the highest median RMSSD, the difference between the medium- and high-PA groups was not significant (*P* = NS).

Mean RR interval did not differ significantly among the three PA groups (all pairwise *P* = NS).

Frequency-domain measures:

Normalized frequency-domain parameters (low frequency component (LFn), high-frequency component (HFn), and low frequency/high frequency (LF/HF) ratio) showed no significant between-group differences (all *P* = NS). Although the low-PA group exhibited a slightly higher median LF/HF ratio compared with the high-PA group, this trend was not statistically significant.

### Correlation analysis: HRV with physical activity, body composition, and cardiopulmonary fitness

**Figure 3:**
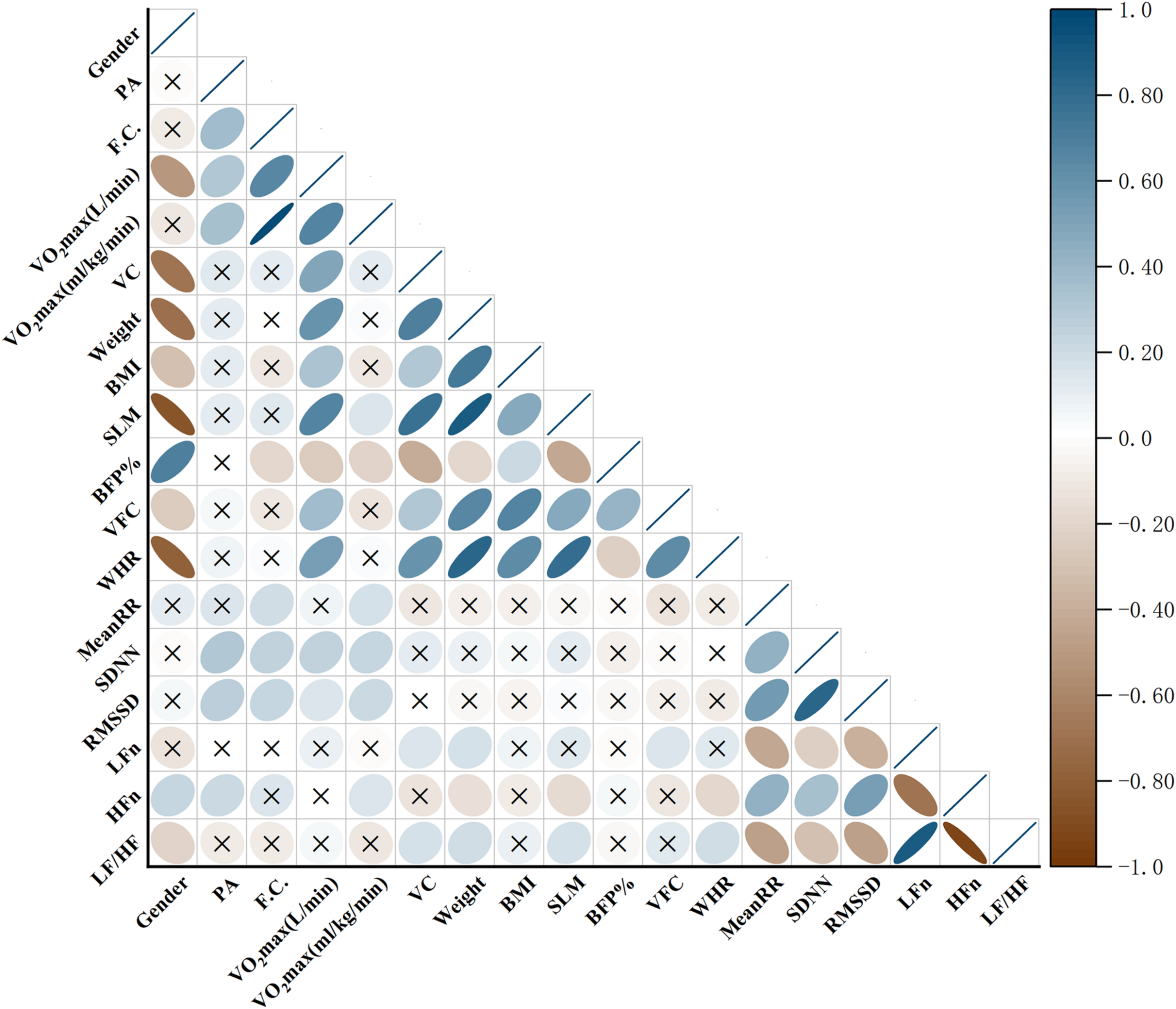
Heatmap of correlations among physical activity, cardiorespiratory fitness, body composition, and HRV indices. Heatmap illustrating the correlations among physical activity (PA), cardiorespiratory fitness variables (functional capacity, vital capacity, and VO_2_max), body composition indices (BMI, SLM, BFP%, VFC, and WHR), and heart rate variability (HRV) indices (MeanRR, SDNN, RMSSD, LFn, HFn, and LF/HF). The color scale indicates the direction and magnitude of the correlations, with blue denoting positive and brown denoting negative associations. Non-significant correlations (*P* ≥ 0.05) are marked with “×”.

The associations between physical activity, cardiorespiratory fitness, body composition, sex, and HRV indices are summarized in Fig. 3.

1. Physical activity level and HRV
Spearman correlation analysis revealed significant associations between PA level and time-domain indices of HRV. Specifically, PA was positively correlated with SDNN (r = 0.313, *P* < 0.0001) and RMSSD (r = 0.277, *P* = 0.0007), indicating that individuals with higher PA levels tended to exhibit greater overall HRV. In addition, PA showed a medium positive correlation with the normalized high-frequency component (HFn) (r = 0.209, *P* = 0.0138), reflecting a potential relationship between higher PA levels and greater parasympathetic modulation. These findings suggest that higher PA levels are associated with more favorable autonomic profiles.
2. Cardiorespiratory fitness and HRV
Cardiorespiratory fitness showed positive associations with several HRV indices. Specifically, MeanRR was positively correlated with functional capacity (r = 0.186, *P* = 0.0309) and relative VO_2_max (r = 0.176, *P* = 0.0433). SDNN was positively correlated with functional capacity (r = 0.249, *P* = 0.0028), absolute VO_2_max (r = 0.248, *P* = 0.003), and relative VO_2_max (r = 0.231, *P* = 0.0057). Similarly, RMSSD was positively correlated with functional capacity (r = 0.231, *P* = 0.0057) and relative VO_2_max (r = 0.207, *P* = 0.0147). These findings indicate that individuals with higher CRF levels tend to exhibit greater HRV.
3. Body composition, sex, and HRV
Associations between body composition, sex, and HRV were primarily observed in frequency-domain parameters. Specifically, female sex was positively correlated with HFn (r = 0.229, *P* = 0.0062). In contrast, sex was negatively correlated with the LF/HF ratio (r = −0.209, *P* = 0.0139), further supporting sex-related differences in autonomic modulation.

Regarding body composition, WHR was negatively correlated with HFn (r = −0.195, *P* = 0.0236), indicating that abdominal fat accumulation may be associated with reduced parasympathetic activity. Conversely, WHR was positively correlated with the LF/HF ratio (r = 0.187, *P* = 0.0303), suggesting a relative predominance of sympathetic modulation. Other body composition indices, including body weight (r = 0.183, *P* = 0.0339), skeletal muscle mass (SMM) (r = 0.175, *P* = 0.0449), and forced vital capacity (FVC) (r = 0.172, *P* = 0.0499), were all positively correlated with the LF/HF ratio.

### Multiple regression analysis of HRV

**Figure 4:**
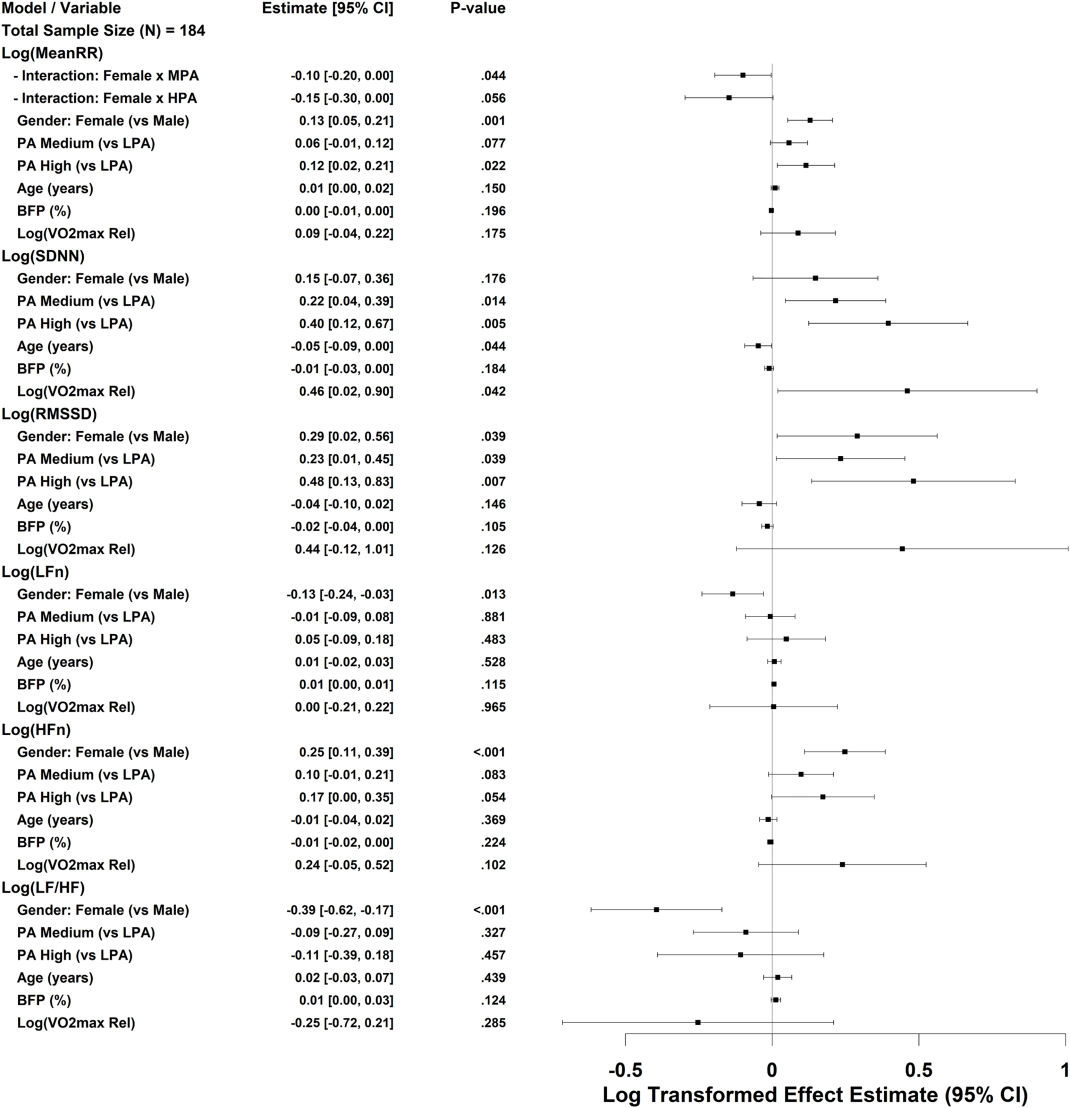
Forest plot of multivariable regression analysis for HRV indices. Forest plot illustrating the results of stepwise multiple regression analyses examining the associations of HRV indices with physical activity, BFP%, and sex. All HRV variables were log-transformed prior to modeling. Relative maximal oxygen uptake (VO_2_max) was also log-transformed and is denoted as log VO_2_max_rel. Regression coefficients are shown with 95% confidence intervals. A *P*-value < 0.05 was considered statistically significant.

A multivariable linear regression model was constructed, with the full model including heart rate variability, sex, age, BFP%, and relative maximal oxygen uptake (logVO_2_max_rel) as potential covariates (Fig. 4). To reduce skewness and meet the assumptions of linearity, all HRV outcome variables were log-transformed. The final model retained the main effects and incorporated an interaction term between sex and PA.

1. physical activity and sex
Compared with the low PA group (LPA), both the medium and high PA groups (MPA and HPA) were significantly associated with higher time-domain HRV indices. Specifically, HPA independently predicted higher log SDNN and log RMSSD values (b = 0.40 and 0.48, respectively), while MPA also showed a positive but smaller effect. These findings indicate a dose–response relationship between PA level and HRV.
After adjusting for age, body composition, and CRF, females exhibited significantly higher values in log MeanRR, log RMSSD, and log HFn, and significantly lower values in log LFn and log(LF/HF) compared with males, suggesting sex-specific differences in autonomic balance. The sex × PA interaction term was significant only in the log MeanRR model (Female × MPA), while interaction terms in other models did not improve AIC values and were thus excluded from the final analysis.
2. Body composition and age
BFP% did not significantly predict any HRV indices. Age was mildly but significantly negatively associated with log SDNN (b = −0.05, *P* = 0.044), indicating that, after controlling for other covariates, each additional year of age corresponded to an approximately 5% reduction in SDNN. Other body composition parameters (*e.g*., visceral fat content and waist-to-hip ratio) were not retained in the final model.
3. Cardiorespiratory fitness
Relative maximal oxygen uptake (log VO_2_max_rel) was a significant positive predictor of log SDNN (b = 0.46, *P* = 0.042), but not of other HRV indices. This finding indicates that CRF exerts a modest facilitating effect on overall HRV, particularly in enhancing global autonomic activity, although its independent contribution remains limited. The generalized variance inflation factor (GVIF) results showed low collinearity between PA and CRF (GVIF < 1.35), confirming that this relationship was not confounded by multicollinearity.

### Discussion

The American College of Sports Medicine (ACSM) has indicated that a higher physical activity level is typically associated with greater cardiorespiratory fitness (CRF), and both are linked to a dose–response relationship with reduced risk of premature mortality. Previous studies have separately confirmed that improvements in either PA or CRF can lower the risk of early death (Haskell et al., 2007; Plaza-Florido et al., 2022). In the present study, significant differences in CRF were observed across PA levels among overweight and obese individuals (Fig. 2), and PA was positively correlated with CRF (Fig. 3), suggesting that higher PA tends to accompany greater CRF in this population, which may confer potential health benefits.

While IPAQ-SF may introduce some misclassification, group-level comparisons remain reliable, making it suitable for assessing physical activity in cross-sectional studies. In this study OW and OB college students engaging in medium or high-intensity PA exhibited higher HRV compared with those performing low intensity PA. Both time- and frequency-domain indices were greater in the medium and high PA groups. For instance, SDNN in the high PA group was 31.29 ms, which was 75% higher than that in the low PA group (17.88 ms), indicating a relative advantage in autonomic regulation capacity. Multiple regression analysis further demonstrated that high-intensity PA independently predicted log(SDNN) and log(RMSSD), whereas medium intensity PA showed an independent significant effect on log(SDNN). These findings align with previous literature indicating an association between PA and cardiovascular autonomic function (Grässler et al., 2021; Königstein, Dipla & Zafeiridis, 2023; Lee, Lee & Ha, 2022). Although the high intensity group showed numerically higher HRV values, the differences from the medium group were not statistically significant. Potential mechanisms through which PA improves HRV include anti-inflammatory effects (Barrón-Cabrera et al., 2020), enhancement of endothelial function (Goeder et al., 2024; Tao et al., 2023), and increased parasympathetic tone promoting autonomic balance (Lujan & DiCarlo, 2013; Tornberg et al., 2019).

After adjusting for age and BMI categories, sex differences remained evident. Females showed higher log(HFn) and lower log(LF/HF) compared with males, indicating relatively greater parasympathetic activity. This pattern is consistent with previously reported HRV sex differences in healthy populations (Geovanini et al., 2020; Stuckey et al., 2014), which may be attributed to the modulatory effects of estrogen on autonomic function and inherent differences between sexes in resting heart rate, arterial pressure, and baroreflex sensitivity (Dearing, Handa & Myers, 2022; Virtanen et al., 2000).

Interaction analysis revealed that medium-intensity PA among females had a significant effect on log(MeanRR), while no significant effect was observed in the high-intensity group, possibly due to differences in exercise intensity and sample distribution. Overall, these findings underscore the importance of considering sex as a key factor when evaluating cardiovascular autonomic function in overweight and obese college students, to facilitate the development of personalized intervention strategies.

Higher waist-to-hip ratio and weight were associated with a higher LF/HF ratio, suggesting a possible shift toward greater sympathetic dominance in individuals with higher adiposity. Notably, skeletal muscle mass was positively correlated with LF/HF, consistent with some previous studies suggesting that higher muscle mass contributes to cardiovascular health, typically associated with greater parasympathetic activity (Zheng et al., 2024). However, in the multiple regression model, skeletal muscle mass did not independently predict any HRV indices. Possible explanations include: (1) high collinearity with other predictors; (2) systemic effects of excess adiposity dominating under overweight/obese conditions; and (3) a potentially nonlinear or complex relationship between muscle mass and autonomic function. Future research should further explore these intricate interactions.

Although physical activity reflects individuals’ behavioral engagement, maximal oxygen uptake (VO_2_max) captures the integrated physiological capacity of the cardiovascular, pulmonary, and metabolic systems. These aspects cannot be inferred solely from PA. Evidence indicates that PA and CRF contribute independently to cardiometabolic risk and mortality (Myers et al., 2015). In the present study, the independent associations between VO_2_max and most HRV indices were attenuated after adjusting for PA levels, except for SDNN. This attenuation may reflect overlapping physiological influences of aerobic fitness and moderate-to-vigorous PA on autonomic regulation. Previous studies have reported similar findings, suggesting that PA and VO_2_max share substantial physiological variance in explaining HRV variability (Tangen et al., 2022). Notably, our study population consisted of overweight and obese university students. In this group, HRV appeared to be more strongly associated with PA than with VO_2_max in multivariable models. This may indicate that, in this specific population, individual VO_2_max is constrained by body composition and metabolic factors, whereas medium-to-high PA is more closely related to HRV. Finally, although the Åstrand-Rhyming submaximal test is more cost-effective and practical than laboratory-based direct VO_2_max measurement and has been widely applied in obese populations, its accuracy may be limited in this group. Body weight and heart rate responses can introduce estimation errors, particularly in individuals with higher BMI (Dearing, Handa & Myers, 2022).

The relatively low R^2^ values of the regression models suggest that HRV among overweight and obese college students is influenced by numerous endogenous and exogenous factors, many of which cannot be fully captured in a single cross-sectional assessment. A recent review classified potential confounders of HRV into four broad categories: (1) physiological factors (*e.g*., age, sex, genetics, circadian rhythms, menstrual cycle), (2) disease-related factors (*e.g*., cardiometabolic disorders, psychiatric conditions), (3) lifestyle and behavioral factors (*e.g*., sleep quality, chronic stress, smoking, alcohol and caffeine intake, medication use), and (4) measurement-related factors (Sammito, Thielmann & Böckelmann, 2024). Future studies should consider these influences and adopt longitudinal or interventional designs to better elucidate causal relationships.

### Limitations

This study has several limitations. First, although a 2-week open monitoring period was used before testing, the self-reported PA intensity data may still be subject to recall bias and social desirability bias, and some selection bias may remain. The high-intensity group also had a relatively small sample size (*n* = 21), so comparisons involving this group should be interpreted cautiously. In addition, for female participants, menstrual cycle phase was not recorded at the time of HRV assessment and may represent an unmeasured confounder. Finally, the cross-sectional design limits the ability to draw causal inferences.

## Conclusion

Medium-to-high physical activity was found to independently predict specific heart rate variability indices in overweight and obese college students. Moreover, female students exhibited higher values in multiple HRV parameters compared with their male counterparts, and these sex differences remained significant after adjusting for other covariates. Notably, the independent predictive effect of cardiorespiratory fitness on HRV appeared weaker than that of PA in this population.

## Supplemental Information

10.7717/peerj.20612/supp-1 Supplemental Information 1 The raw measurements.

10.7717/peerj.20612/supp-2 Supplemental Information 2 Codebook.

10.7717/peerj.20612/supp-3 Supplemental Information 3 STROBE checklist.

## Additional Information and Declarations

### Data Availability

The following information was supplied regarding data availability:

The raw data is available in the Supplemental File.
