# Supplementary material for: The impact of physical activity levels and cardiorespiratory fitness on heart rate variability in overweight and obese college students: a cross-sectional study
Source: PeerJ. 2026 Jan 19;14:e20612. doi: 10.7717/peerj.20612 (PMC12826037; doi:10.7717/peerj.20612)
Supplement: Supplemental Information 2 [file peerj-14-20612-s002.docx]

| **Column Name** | **Meaning** |
| --- | --- |
| **Name** | Participant Identifier |
| **Gender Biological Sex** | 1 = Male, 2 = Female |
| **BMI grouping (BMI) Classification** | 1 = Overweight, 2 = Obese |
| **PA Physical Activity Level** | 1 = Low Activity, 2 = Moderate Activity, 3 = High Activity |
| **HRV Metrics** | MeanRR,SDNN,RMSSD,LFn,HFn,LF/HF |
| **Body Composition Metrics** | SLM,BFP%,VFC,WHR |
| **Cardiopulmonary Function Metrics** | VO2max(L/min),VO2max(ml/kg/min),VC |
| **Total activity value** | IPAQ-Short Form score |
